# Molecular Recognition by Silicon Nanowire Field-Effect Transistor and Single-Molecule Force Spectroscopy

**DOI:** 10.3390/mi13010097

**Published:** 2022-01-08

**Authors:** Francisco M. Espinosa, Manuel R. Uhlig, Ricardo Garcia

**Affiliations:** Instituto de Ciencia de Materiales de Madrid, Consejo Superior de Investigaciones Cientificas, c/ Sor Juana Inés de la Cruz 3, 28049 Madrid, Spain; francisco.espinosa@csic.es (F.M.E.); manuel.uhlig@csic.es (M.R.U.)

**Keywords:** oxidation scanning probe lithography, silicon nanowire, field-effect transistor, single-molecule force spectroscopy, AFM

## Abstract

Silicon nanowire (SiNW) field-effect transistors (FETs) have been developed as very sensitive and label-free biomolecular sensors. The detection principle operating in a SiNW biosensor is indirect. The biomolecules are detected by measuring the changes in the current through the transistor. Those changes are produced by the electrical field created by the biomolecule. Here, we have combined nanolithography, chemical functionalization, electrical measurements and molecular recognition methods to correlate the current measured by the SiNW transistor with the presence of specific molecular recognition events on the surface of the SiNW. Oxidation scanning probe lithography (o-SPL) was applied to fabricate sub-12 nm SiNW field-effect transistors. The devices were applied to detect very small concentrations of proteins (500 pM). Atomic force microscopy (AFM) single-molecule force spectroscopy (SMFS) experiments allowed the identification of the protein adsorption sites on the surface of the nanowire. We detected specific interactions between the biotin-functionalized AFM tip and individual avidin molecules adsorbed to the SiNW. The measurements confirmed that electrical current changes measured by the device were associated with the deposition of avidin molecules.

## 1. Introduction

Different types of field-effect transistor were proposed to detect biomolecular processes [1,2,3,4,5]. Among them, silicon nanowire FETs offered a genuine nanoscale platform to detect biological molecules. Those devices have targeted different biomedical and biological applications as the specific detection of viruses, proteins, nucleic acids, cancer biomarkers, and cells or monitoring the physiological responses of a specific therapeutic treatment on cells or tissues [6,7,8].

Top-down approaches such as scanning probe lithography were applied to fabricated SiNW devices [9,10,11]. In particular, oxidation scanning probe lithography (o-SPL) was applied to fabricate biosensors [12]. Oxidation SPL combines the high-spatial resolution capabilities of atomic force microscopy (AFM) for positioning with the capability to fabricate nanoscale features [13,14]. It has been extensively applied in nanopatterning and for the fabrication of a variety of nanoelectronics devices [10,11,12,13,14,15,16,17,18,19,20,21,22,23,24,25,26,27,28,29].

Here, oxidation SPL, silicon nanowire transistors and single-molecule force spectroscopy are combined to quantify the molecular recognition events happening on the surface of a silicon nanowire biosensor. The SiNW field-effect transistor detected very low concentrations of proteins (500 pM). We demonstrated the existence of specific biomolecule interactions on the surface of the SiNW. Those interactions were associated with the presence of avidin molecules. The study aimed to bridge the gap between the indirect measurements of biomolecular activity provided by nanoscale transistors and the direct measurements provided by single-molecule force spectroscopy.

## 2. Materials and Methods

### 2.1. Silicon Nanowires

The silicon nanowires were made by o-SPL on substrates from ultra-thin silicon on insulator (SOI) wafer (MEMC/SunEdison, Belmont, CA, USA). The top (100)-oriented Si layer is 12 nm thick, p-doped, has a nominal resistivity of 9–15 Ω cm and the buried oxide layer (BOX) has 25 nm of thickness. The substrates were cleaned with a protocol that involves three sonication cycles of 10 min each in a mixture of NH_4_OH–H_2_O_2_–H_2_O (1:1:5). Then, the samples are sonicated for 5 min in deionized water.

Oxidation SPL was applied to generate ultrathin and narrow silicon oxide masks. It was performed at a relative humidity of 45%. We have used n +-type doped silicon cantilevers (NCHV-W, Bruker) with a force constant of about 42 N m^−1^ and a resonant frequency of 320 kHz. The cantilever was excited at its resonant frequency. Typical voltage pulses were of 21–27 V with 0.7–1 ms duration. General and detailed aspects about o-SPL might be found elsewhere [14]. The SiNWs used here were 10–12 nm in thickness, 150 nm in width and 5 µm in length.

In a later stage, the ultrathin oxide mask was removed by applying reactive ion etching (NRE 300 RIE System de NANO-MASTER, Inc, Austin, TX, USA). The sample was introduced in the RIE chamber and the pressure lowered to 10^−5^ Torr for 30 min. Then the SF_6_–O_2_ gas mixture (10:5 sccm) was introduced and left to stabilize for 1 min at the specific chamber pressure of the experiment (6.25 × 10^−2^ Torr). A 20 W radio frequency power was applied for 45 s.

### 2.2. Microelectrodes

First, a layer of photoresist S1813 was deposited on the sample and centrifuged at 5000 rpm for 1 min. It was cured on a hot plate (115 °C) for 1 min. The microelectrodes were patterned by UV photolithography. A UV mask was used to protect the transistor channel from light exposure (8 s). Then, the sample was immersed in the developer (MF319) for 1 min, rinsed with deionized water and dried with N_2_. After that, the sample was treated with oxygen plasma for 30 s to eliminate the resist residues. To make the microelectrodes, a thin film Cr (5 nm)/Au (40 nm) was deposited by electron beam evaporation.

The electrodes were protected from the buffer by a thin film of polydimethylsiloxane (PDMS). A solution of PDMS and hexane (1:100) was centrifuged at 3000 rpm for 30 s. The resulting thickness was about 30 nm. The sample was cured on the hotplate for 30 min at 90 °C. In a later stage, the resist covering the channel was removed with acetone and rinsed with isopropanol and water. An O_2_ plasma was used to remove the possible remains of organic residues from the surface of the microfluidic channel. To generate the O_2,_ a power of 50 W was applied for 1 min in a chamber with a pressure of 0.5 mbar (0.37 Torr).

### 2.3. Chemicals

Phosphate-buffered saline (PBS) powder, ethanol, 30% hydrogen peroxide, 1 N (0.5 mol/L) sulfuric acid, 3-aminopropyltriethoxysilane (APTES) 99%, trimethylamine ≥ 99% and avidin from egg white ≥ 98% were purchased from Sigma-Aldrich (Madrid, Spain). The N-Hydroxysuccinimid (NHS)-polyethylenglycol (PEG27) biotin linkers were purchased from JKU Linz [30].

### 2.4. Samples Silanization with OTS

Before octadecyltrichlorosilane (OTS) functionalization, the samples were exposed to an oxygen plasma. The conditions of the process were: 50 W, 0.4 mbar and 30 s. The samples were immediately immersed in 99.5% anhydrous toluene. In a glove box with an N_2_ atmosphere, the samples were passed from anhydrous toluene to a 5 mL solution of anhydrous toluene at 99.9% with 3 μL of OTS for 105 s. Then, the sample was rinsed with chloroform, ethanol and water, and dried with nitrogen. The sample with the OTS was cured on a hot plate at 80 °C.

### 2.5. Proteins

A freeze-dried avidin was dissolved in 1 mM NaCl to obtain a solution with an avidin concentration of 500 pM. Then, 100 µL of the solution was deposited on the SiNW sample for 1 min. Afterwards, the sample was rinsed carefully with 1 mM NaCl and subsequently with 20 mM PBS.

### 2.6. Tip Functionalization with APTES

Silicon nitride cantilevers (MSCT, Bruker, Camarillo, CA, USA) were cleaned thoroughly using the RCA procedure. This procedure consisted of three subsequent baths in a mixture of ammonia solution, hydrogen peroxide (30%) and ultrapure water (1:1:5 ratio in volume). Then, the cantilevers were rinsed with ultrapure water and dried carefully using a flow of N_2_. Afterwards, the cantilevers were exposed to oxygen plasma (Diener Electronic, Ebhausen, Germany) for 1 min at a power of 100 W under a pressure of 0.4 bar. Next, the cantilevers were transferred into a mixture of 3-aminopropyltriethoxysilane (APTES) and ethanol (1:5000 ratio in volume) to functionalize the tips. After 45 min, the cantilevers were rinsed with ethanol and ultrapure water and dried with N_2_. Finally, the cantilevers were placed in a desiccator for 1 h.

### 2.7. Tip Functionalization with PEG-Biotin

The functionalization of the tips with NHS-PEG27-Biotin linkers followed a similar protocol as described above. First, 1 mg of the NHS-PEG27-Biotin linkers was dissolved in trichloromethane (0.5 mL). The obtained solution was transferred into a Teflon PTFE chamber and 30 µL of tryethylamine were added as a catalyst. Then, the silanized tips were immersed into the chamber. After an incubation time of 2 h, the cantilevers were removed from the chamber, rinsed three times with trichloromethane and dried with N_2_.

### 2.8. Cantilever Calibration

Cantilevers of the types PPP-NCH-W (Si, NanoWorld) and MSCT-C (SiN_x_, Bruker) were used for AFM imaging. For the SMFS experiments, we used MSCT-D cantilevers (SiN_x_, Bruker). MSCT-D cantilevers were calibrated in liquid as follows: First, the inverse optical lever sensitivity (invOLS) for the static detection, σ, was obtained from force-displacement curves recorded on the gold electrodes. The invOLS σ was determined as the inverse of each curve’s slope in the contact part and then averaged over 256 curves. Second, the cantilever’s thermal noise spectrum (power spectral density, PSD) was recorded at about 15 µm above the sample surface [31]. Then, the single harmonic oscillator (SHO) model was fitted to the PSD around the peak of the first resonance frequency using the calculated invOLS of the first mode, *σ*_1_ [32]. The fitting yields the force constant *k*_1_. The static force constant can then be calculated by *k*_0_ = *k*_1_/1.03. The obtained values for the cantilevers used here were *k*_0_ = 32 pN/nm (Experiment 1) and *k*_0_ = 39 pN/nm (Experiment 2). The calibration was performed after the experiment to prevent tip damage during the calibration.

### 2.9. AFM Experiments

All the experiments were performed on a NanoWizard III AFM (JPK Instruments AG, Berlin, Germany) equipped with an open liquid cell. The experiments were carried out in 10 mM PBS at pH 7.4 at a temperature of *T* = 302 K. First, the SiNW was localized by AFM using an MSCT-C cantilever. Then, the position of the SiNW was marked in the optical image from the camera, and the laser spot was moved to the MSCT-D cantilever on the same chip. The new cantilever was then moved to the previously marked position and an SMFS measurement was started without further imaging. This procedure was developed to prevent damage of the tip functionalization due to AFM imaging. SMFS experiments were performed in the force-volume mode [33]. This method enabled us to directly correlate the obtained curves with the topography of the SiNW. The individual curves were acquired by applying a periodic trapezoidal modulation to the cantilever base with an amplitude of *A*_p_ = 150 nm. First, the cantilever base was brought towards the sample at a constant speed of 200 nm/s until a force of 140 pN was reached. This force value was chosen to avoid damage of the biotin. At this extension, the piezo was held for 200 ms. Then, the cantilever base was retracted at a constant pulling speed of *v*_p_ = 1000 nm/s. FV images were performed on different SiNWs and covered different areas. For experiment 1 (2), it consisted of 20 × 40 (40 × 60) force curves in an area of 0.5 × 1.0 µm^2^ (1.0 × 1.5 µm^2^), resulting in a total of 800 (2400) force curves.

### 2.10. Single-Molecule Force Spectroscopy (SMFS) Analysis

All force-displacement curves were transformed into force-distance curves by *d* = *z* + *F*/*k*. To detect the specific unbinding events, a custom written code was used (MATLAB, MathWorks, Natick, MA, USA) [34]. It fitted the retraction part of the SMFS curves with a polynomial function of grade 7. Then, the minima of the polynomial were determined and the algorithm searched in their vicinity for minima in the raw data. Minima with a force value smaller than 1.5-fold of the baseline noise were disregarded to avoid spurious peaks. Specific unbinding events were selected by applying general criteria for specificity [35,36]. Only events that showed rupture distances *d*_rup_ in the 5 to 17 nm range were considered. The above distance range matched the length of the PEG27 linker (10 nm ± 5 nm for the slightly shorter PEG24 [35]). In total, <2% of the retraction curves exhibited features of specific unbinding events. Curves showing specific unbinding events were further processed using the JPK Data Processing software. The software fitted a freely jointed chain (FJC) model to each curve exhibiting an unbinding event.

(1) $$d\left(F\right)=L_{C}\left[\coth\left(\frac{Fl_{K}}{k_{B}T}\right)-\frac{k_{B}T}{Fl_{K}}\right]$$

where *L*_C_ is the contour length, *l*_K_ = 700 pm the Kuhn length, *k*_B_ the Boltzmann constant and *T* the absolute temperature [37,38]. Furthermore, for each individual unbinding event, the software read out the unbinding force *F*_m_ and determined the loading rate *r* by fitting the force versus time data just before the event.

## 3. Results and Discussion

### 3.1. Fabrication of a SiNW Biosensor

**Figure 1:**
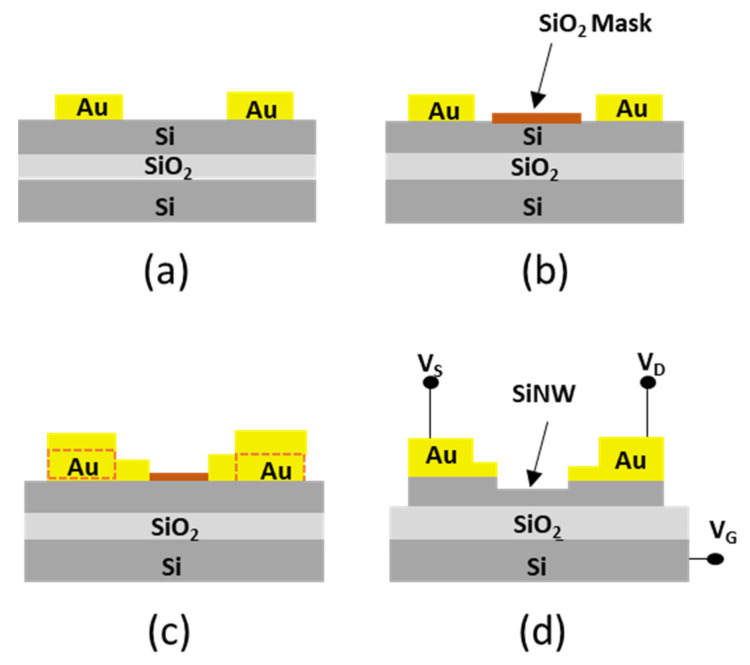
Main steps in the fabrication of a SiNW field-effect transistor by o-SPL. (**a**) Gold electrodes were defined by photolithography and electron beam evaporation. (**b**) Ultrathin and narrow oxide mask made by o-SPL. (**c**) Second photolithography step to connect the Au electrodes and the ultrathin oxide mask. (**d**) Reactive ion etching to remove the unprotected silicon regions. The etching also removed the ultrathin oxide mask. The final result was a back-gated Si nanowire FET.

Figure 1 shows a scheme of the main steps to generate a SiNW field-effect transistor. First, gold microelectrodes were patterned by photolithography on a silicon-on-insulator sample (Figure 1a); o-SPL was applied to define a narrow and ultrathin silicon oxide mask (Figure 1b). A second photolithography step was applied to deposit a thin Au film and to close the gap between the microelectrodes and the o-SPL mask (Figure 1c). The next step involved the etching of the unmasked silicon region by reactive ion etching. Finally, the three electrodes of the back-gated device were connected to power sources (Figure 1d).

**Figure 2:**
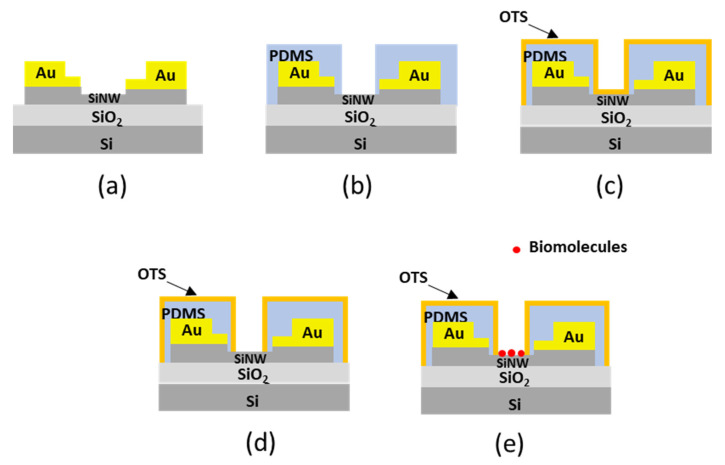
Steps to develop a SiNW biosensor. (**a**) SiNW FET. (**b**) PDMS deposition to protect the Au electrodes from the buffer solution. (**c**) Coating the device with an OTS layer. (**d**) Removal of the OTS from the surface of the SiNW by the AFM tip. (**e**) Adsorption and detection of biomolecules.

We developed a protocol for the selective deposition of the biomolecules on the SiNW surface. The process was based on controlling the adhesion force between the different surfaces and the proteins by using chemical functionalization and controlling the pH of the buffer [39]. The Au electrodes of the SiNW transistor (Figure 2a) were coated with a thin film of polydimethylsiloxane (Dow Corning’s Sylgard Elastomer 184, purchased from Sigma Aldrich). This film was used to separate the Au electrodes from the solution (Figure 2b). Then, an OTS monolayer was deposited over the whole device (Figure 2c). The purpose of this layer was to avoid the unspecific binding of the proteins to the different surfaces of the device. The OTS monolayer was later removed from the SiNW surface by applying a large force with the AFM tip (~2 μN) (Figure 2d). Finally, the proteins (avidin) were deposited (Figure 2e).

**Figure 3:**
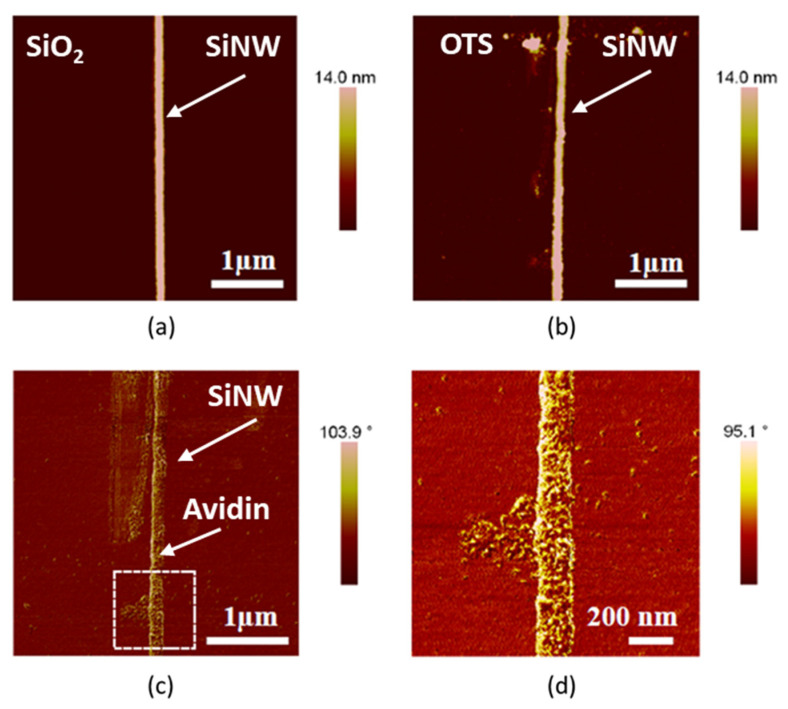
(**a**) AFM topographic image of a SiNW. (**b**) AFM topography of the same SiNW after OTS functionalization and removal. (**c**) AFM phase image after avidin molecules were deposited on the surface of the SiNW. (**d**) High resolution image of the area marked in (**c**). In this experiment, a 100 μL drop containing avidin was deposited over the SiNW for 1 min. Afterwards, the sample was rinsed with a 1 mM NaCl solution and, subsequently, with 0.02M PBS.

Amplitude modulation AFM images of the surface of a SiNW were acquired before (Figure 3a) and after OTS functionalization and removal (Figure 3b). AFM phase images [33] obtained after the deposition of avidin revealed that the proteins were deposited on or near the SiNW surface (Figure 3c,d). From Figure 3d we were able to count the number of avidins over the nanowire. On average, one avidin molecule was found every 5 nm along the SiNW. An avidin molecule occupied approximately 30 nm^2^. In total, we estimated 10,000 molecules over the SiNW shown in Figure 3c.

### 3.2. Electrical Characterization of SiNW

**Figure 4:**
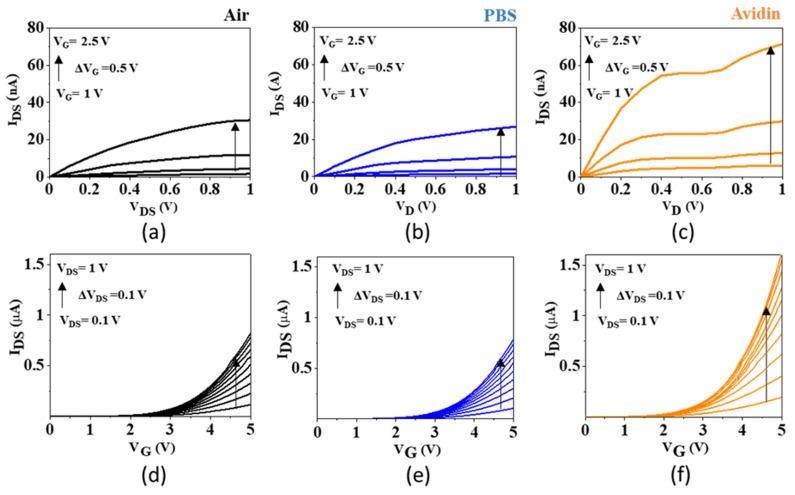
(**a**) Output curves of a SiNW transistor in air. (**b**) Output curves of the same transistor immersed in PBS. (**c**) Output curves of the same transistor after immersion in a liquid containing avidin molecules. (**d**) Transfer curves obtained from (**a**). (**e**) Transfer curves obtained from (**b**). (**f**) Transfer curves obtained from (**c**).

Figure 4a,b showed the output curves of a SiNW FET in air and after its immersion in PBS (control measurements). The curves were very similar in both environments. This experiment showed the stability of the device in a liquid environment. Figure 4c showed the response of the same SiNW after a drop of a liquid containing avidin molecules was drop-casted. A significant increase of the current was observed (Figure 4c) with respect to the curves obtained in air or PBS. The increase of the current was consistent with the positive charge carried by avidin (the pH of the buffer was 7.4 while the isoelectric point of avidin was about 10). The corresponding transfer curves are shown in Figure 4d–f.

### 3.3. Single-Molecule Force Spectroscopy on a SiNW

**Figure 5:**
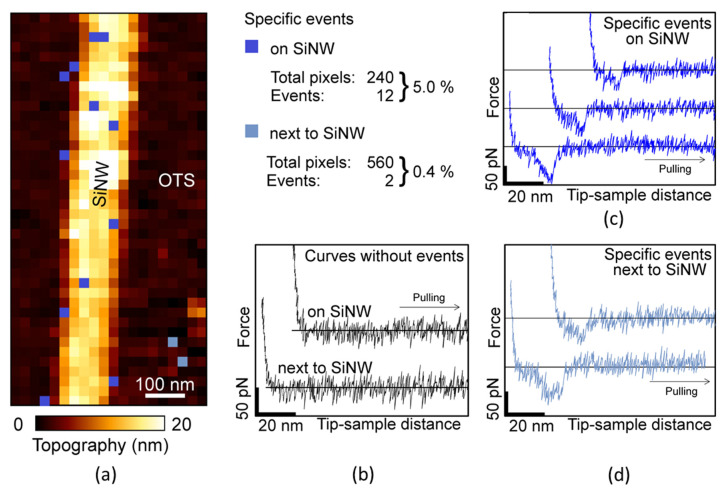
Detection of specific unbinding events on a silicon nanowire surrounded by an octadecyltrichlorosilane (OTS) layer. (**a**) AFM topography image obtained over the SiNW by force volume imaging. Each pixel corresponds to an entire force-distance curve. Pixels where specific avidin-biotin events were detected were marked in blue tones (blue: on the SiNW, light blue: next to the SiNW). (**b**) Examples of force-distance curves without unbinding events. (**c**) Examples of force-distance curves with specific unbinding events taken on the SiNW. (**d**) Force-distance curves with specific unbinding events taken on the OTS layer.

Figure 5a shows the topography maps obtained from the SMFS force volume image. The image shows the SiNW in the center, surrounded by the OTS-covered substrate. Each pixel corresponds to an individual force-distance curve. While most of the retraction curves were featureless (Figure 5b), some curves exhibited a specific unbinding event. The positions of these curves are marked in blue. Light and dark blue corresponds to the OTS-covered substrate and the SiNW, respectively. Some example curves with specific unbinding events are shown in Figure 5c,d.

In total, 14 out of 800 force-distance curves showed specific events, from which 12 were located on the SiNW or right next to it. The SiNW had a width of approximately 6 pixels in the image, and consequently covered an area equivalent to 240 pixels. Thus, 240 force-distance curves were obtained on the SiNW. Hence, on the SiNW, the chance to obtain a specific unbinding event was 5.0% (12/240), while on the surface covered by OTS it was <0.4% (2/560). This result showed that the functionalization protocol significantly increased the chance for a target molecule (avidin) to be deposited on the SiNW’s surface. The overall rate of recognition (5.0%) was lower than some values reported elsewhere (around 27–29%) [30,40,41]. The difference might be explained by the degrees of immobilization of the avidin. In previous works, the avidin was physisorbed to mica while here was bound to the SiNW. The stronger binding resulted in a lower flexibility of the avidin and hence reduced the probability that the biotin was bound to one of the avidin’s four binding sites during the moment of contact. In any case, the key result here was that the binding probability found on the SiNW was one order of magnitude larger than the one found on the surfaces covered by OTS. A second experiment performed on a different SiNW confirmed the above finding, albeit the selectivity was slightly lower (5.7% on the SiNW versus 1.0% on the OTS-covered substrate).

#### Bell–Evans Model

**Figure 6:**
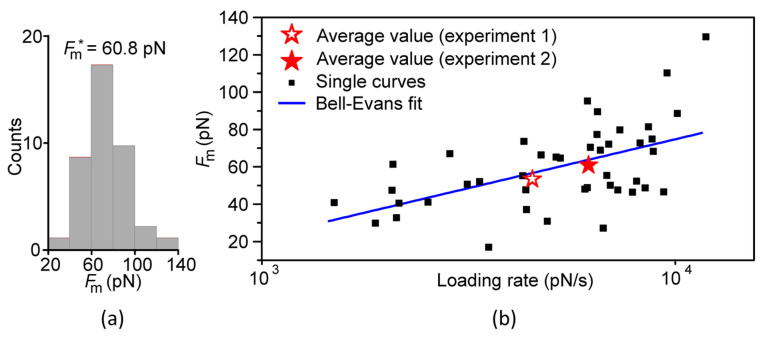
Quantitative analysis of the observed unbinding events. (**a**) Histogram of the measured forces *F_m_* with an average value of F_m_* = 60.8 pN (data from experiment 2). (**b**) Measured forces versus the loading rate. The data from all individual curves was shown (black squares) as well as the averaged values (red stars). The Bell–Evans fit including all black squares was shown in blue. The fit parameters were x_β_ = (0.21 ± 0.06) nm and *k*_off_ = (21.4 ± 14.1) s−^1^.

In order to verify the specificity of the observed unbinding events we applied the Bell–Evans model [42,43]. Figure 6a shows the overall distribution of force values. The average measured force, F_m_* = 60.8 pN, agreed well with reported data obtained with cantilevers of similar stiffness [34,40].

A ligand–receptor system characterized by the presence of weak non-covalent bonds exhibits a dependence of the rupture forces (bond strengths) on the loading rate at which the force is applied [41]. The Bell–Evans theory provides the basic understanding of this dependency [42,43]. With increasing loading rate *r*, the mean value of the rupture force *F*_rup_ increased according to:

(2) $$F_{\mathrm{rup}}\left(r\right)=-\frac{k_{B}T}{x_{u}}\ln\left(\frac{rx_{u}}{k_{\mathrm{off}}k_{B}T}\right)$$

where *k*_off_ is the dissociation constant, *x*_u_ the effective distance between the bound and unbound states, *T* the absolute temperature and *k*_B_ the Boltzmann constant. Figure 6b showed the data from two experiments performed under similar conditions. The loading rate *r* depended on both the pulling speed *v*_p_ and the effective linker stiffness, *k*_eff_ according to *r* = *v*_p_ *k*_eff_. The effective linker stiffness was related to the stiffness of the PEG-linker, *k*_PEG_, and the cantilever stiffness, *k*_0_, by $k_{\mathrm{PEG}}=\frac{k_{0}k_{\mathrm{eff}}}{k_{0}-k_{\mathrm{eff}}}$.

Although the experiments were performed using a constant pulling speed *v*_p_, the loading rate covered a range of values for two reasons. First, the cantilevers used for the two experiments had different stiffnesses *k*_0_. Second, the stochastic nature of the unbinding process provided a range of rupture lengths, which, for a nonlinear spring, implied a distribution in the PEG stiffness [37].

The measured average force values were represented by the red stars. In order to increase the fit quality, it was useful to plot the individual data points instead of the average ones [44,45,46]. Fitting all data points using Equation (2) described the data well (both the average force values and the individual ones). The fit yielded x_β_ = (0.21 ± 0.06) nm and *k*_off_ = (21.4 ± 14.1) s ^−1^ which was close to the values obtained previously for the inner activation barrier [34,40].

Equation (2) was based on Hooke’s law, which implies an accuracy limit for its application in AFM-based SMFS [47]. The validity of Equation (2) was verified by calculating the frequency ratio χ by [34].

(3) $$\chi =\frac{v_{p}}{f_{1}x_{c\text{-}c}}$$

For the measurement conditions used here, we obtained $\chi =\frac{1000\;\mathrm{nm}/s}{3000\;s^{-1}\;0.15\;\mathrm{nm}}\approx 2.2$. Such a value of χ implied that the dynamic terms were very small and neglecting them led to an error of less than 1%. Hence, the unbinding data was described accurately by Equation (2) (Bell–Evans model).

Altogether, it was concluded that the observed features were signatures of specific biotin-avidin unbinding events. The above results confirmed that the functionalization protocol of the SiNW provided a selective adsorption site for avidin molecules.

## 4. Conclusions

We aimed to correlate the indirect measurements of biomolecule concentrations obtained by SiNW biosensors with the direct molecular recognition data provided by single-molecule force spectroscopy. To that end, we applied oxidation nanolithography, surface functionalization, electrical measurements and single-molecule force spectroscopy. A liquid containing avidin molecules increased the current through the SiNW device with respect to the control experiments. This was consistent with the positive charge carried by avidin. The SiNW field-effect transistor detected very low concentrations of avidin molecules (500 pM). In a subsequent experiment, we demonstrated the existence of specific molecular recognition events associated with the presence of avidin on the surface of the SiNW biosensor. Therefore, we confirmed that the nanoscale transistor measurements were correlated with the presence of avidin biomolecules on the surface of the nanowire.
